# Anterior saggital anorectoplasty and peri-vaginal musculature

**DOI:** 10.4103/0971-9261.57708

**Published:** 2009

**Authors:** Dinesh Kittur

**Affiliations:** Department of Pediatric Surgery, Ankur Hospital for Children, 1666, E, 10^th^ Lane, Rajarampuri, Kolhapur, India

Sir,

While performing an anterior sagittal anorectoplasty (ASARP) we are usually concerned about the proper site, the size of the neo-anus, but reconstruction of vaginal muscles seems to be ignored.

Following are the important points to be considered regarding the anatomy of the pelvic diaphragm:

1. Disposition of iliococcygeus, ischiococcygeus and pubococcygeus is in the form of a cone-shaped basin.
2. In the female, the innermost and lowermost fibers of this diaphragm encircle the three important structures, that is, urethra, vagina in its lower one-third and the rectum.
  It is known that a tear in the perineal body may cause divarication of the levators and contribute to uterovaginal prolapse. This is prevented by an episiotomy, which provides a clean, easily repairable wound.
3. The muscle fibers from each side meet in the midline and give a sphincteric effect to all the three structures.

If we consider the pathological anatomy of female ARM, especially the rectovestibular fistula and vestibular anus, the innermost fibers of the levator (pubovaginalis), encircle both the vagina and the fistula of anal canal at the perineal level. The fibers of the ischiococcygeus and iliococcygeus are attached to the perineum in the midline.

The rule of nature is “Function follows Form.”

Steps for performing “ASARP”

Step 1. The innermost and lowermost fibers of the levator, that is, the pubovaginalis and puborectalis are dissected from the perineal level free from the rectum and vagina so as to dissect the length of rectum, which is adequate to bring it to the desired anal site.

Step 2. The vertical fibers of the iliococcygeus and ischiococcygeus, which have been split into two halves (right and left), are wrapped around the rectum up to the anal canal so as to effectively straighten the angle of the rectum for proper defecation.

It is worth noting that the lowermost fibers of the pubococcygeus (pubovaginalis), which are supposed to encircle the lower one-third of the vagina are left deep inside, which is approximately 2 cm in small infants and 5-7 cm in older children. Also during the repair of the perineum these are not attached to the newly constructed perineal body.

The whole effect of the above mentioned procedure is that the pelvic diaphragm becomes a wide and shallow cone. Considering “Starling's Law,”[1] the length of the muscle fibers encircling the vagina is reduced since their attachment to the perineal body is not repaired; hence, the function is bound to be hampered in later life.

What are the problems that we can anticipate for these girls in later life during adulthood?

1. The vagina does not have pelvic muscles for support in the lower one-third.
2. These females will not be able to exert a squeezing effect on the penis during coitus.
3. The physiology of orgasm will be disturbed and the propagation of sperms toward the cervix will be altered.
4. These patients will be more prone to prolapse of the uterus.[2]

I feel that we need to consider a few steps during “ASARP” to set the anatomy right and hope to give normal sexual pleasures to these patients in their adulthood.[3] Sexual satisfaction during coitus is very personal and subjective; thus, it is difficult to assess these factors during adulthood in these patients. Such an assessment would evaluate the success of this procedure.
